# Tissue-infiltrating immune cells contribute to understanding the pathogenesis of Kimura disease: A case report

**DOI:** 10.1097/MD.0000000000018300

**Published:** 2019-12-16

**Authors:** Takashi Maehara, Ryusuke Munemura, Mayumi Shimizu, Noriko Kakizoe, Naoki Kaneko, Yuka Murakami, Moriyama Masafumi, Tamotsu Kiyoshima, Shintaro Kawano, Seiji Nakamura

**Affiliations:** a Section of Oral and Maxillofacial Oncology, Division of Maxillofacial Diagnostic and Surgical Sciences; b Department of Oral and Maxillofacial Radiology, Faculty of Dental Science, Kyushu University, Fukuoka, Japan; c Ragon Institute of MGH, MIT and Harvard, Massachusetts General Hospital, Harvard Medical School, Boston, MA; d Laboratory of Oral Pathology, Division of Maxillofacial Diagnostic and Surgical Sciences, Faculty of Dental Science, Kyushu University, Fukuoka, Japan.

**Keywords:** CD4+ T cell, CD4+ GATA3+ T cell, IgE, Kimura disease, mast cell

## Abstract

**Rationale:**

Kimura disease (KD) is a rare, chronic inflammatory disorder characterized by subcutaneous granuloma in the head and neck region, as well as increased eosinophil counts and high serum immunoglobulin E (IgE) levels. Kimura disease is suspected to be an IgE-mediated disease, associated with an allergic response, in which antigen-specific B cells are stimulated to undergo specific IgE class switching with disease-specific CD4+ T (Th) cells help. Thus, exploration of the Th cells in affected tissues with KD is a highly promising field of the investigation. However, there have been no reports with direct evidence to implicate Th cells in affected lesions with KD. Here we quantitatively demonstrate that CD4+ GATA3+ T cells and interleukin (IL)-4+ IgE+ c-kit+ mast cells prominently infiltrate in affected lesion with KD.

**Patient concerns:**

A 56-year-old Japanese man who exhibited painless swelling in the left parotid region.

**Diagnoses:**

Diagnosis of KD was made based on characteristic histopathologic findings, in conjunction with peripheral eosinophilia and elevated serum IgE levels.

**Interventions:**

The patient underwent corticosteroid therapy and had been followed for 2 years.

**Outcomes:**

We report a rare case of KD of the parotid region in a 56-year-old man, followed by corticosteroid therapy for 2 years. The mass decreased in size and skin itchiness decreased after therapy. He was discharged without any complications. Furthermore, we quantitatively demonstrate the dominance of CD4+ GATA3+ T cells in affected tissues of KD and detect IL-4+ IgE+ c-kit+ mast cells in lesions by multicolor staining approaches.

**Lessons:**

The findings from this case suggest that peripheral blood eosinophilia might serve as a marker of recurrent disease, long-term follow-up is necessary due to the possibility of recurrent. Interactions among expanded IgE+ B cells, CD4+ GATA3+ T cells, eosinophils, and activated mast cells might play a critical role in the pathogenesis of KD.

## 1 Introduction

Kimura disease (KD) is a rare, chronic inflammatory disorder that is characterized by subcutaneous granuloma in the head and neck region. Because the etiology of this disease is unknown, there is currently no established preventative management. Affected patients with KD exhibit increased eosinophil counts and high serum immunoglobulin E (IgE) levels. Importantly, KD is thought to be an IgE-mediated allergic disease. Histological analyses have shown that ectopic germinal centers frequently occur in the affected lesions in patients with KD.^[1]^ In extrafollicular foci, B cells that are activated by CD4+ T cells through CD40L may undergo some degree of differentiation into plasma cells and isotype switching.^[2]^ In the follicular/germinal center, specific CD4+ T cells provide help to B cells during T cell-dependent immune responses; they also contribute to isotype switching, germinal center formation, and the selection of high-affinity B cells in the germinal center. In KD, by pathogenic some antigens, antigen-specific B cells are toward to IgE class switching, suggesting that long-lived IgE-producing plasma cells and IgE-producing memory B cells may play an important role in the pathogenesis of this disease.

Exploration of the interactions between B cells and CD4+ T cells is critical to understanding the pathophysiology of KD. The identification of pathogenic T cell clones, as well as characterization of antigens specific to these clones, constitute the first steps in determining the pathogenesis of this disease. Indeed, patients with KD occasionally exhibit clonal proliferation of T cells.^[3]^ However, previous reports have not directly shown CD4+ T cell subsets in affected disease tissues. Although CD4+ T cells are abundant within KD-affected tissue lesions, they have not been investigated in a quantitative manner. Here, we reported a case of KD in the parotid gland (PG) and we used multicolor staining approaches, as previously reported,^[4]^ to investigate directly CD4+ T cells in a lesion from the patient with KD.

## 2 Case report

**Figure 1:**
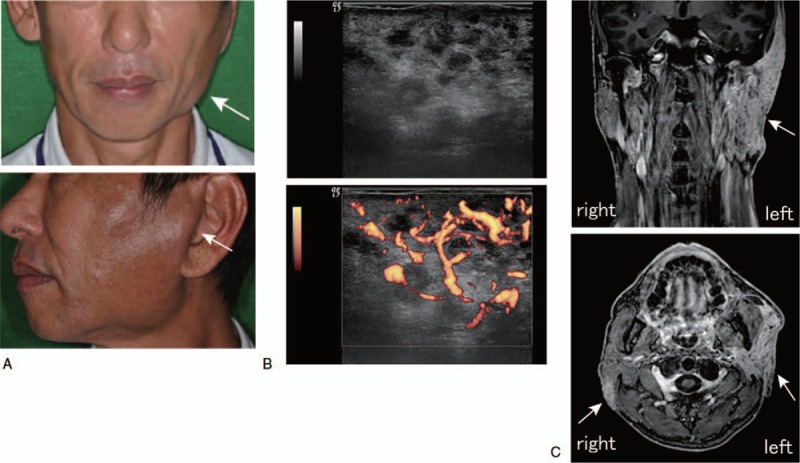
(A) Kimura disease: the patient presented with swelling of left parotid gland. (B) (Upper) Transverse US of the left parotid gland revealed a reticular pattern. (Lower) Power Doppler US revealed enriched vascularity in the gland. (C) MRI T1 weighted image with gadolinium enhancement showed multiple mass lesions (white arrow) involving the swollen left parotid gland and the swollen subcutaneous soft tissues. MRI = magnetic resonance imaging.

A 56-year-old Japanese man had previously attended another hospital with painless swelling of the PGs and right submandibular gland in March 2014. However, he did not receive any treatment and subsequently ignored the swelling; it continued to increase slowly and progressively, without any additional pain. The patient was referred to our institution with painless swelling of the left PG in July 2014 (Fig. 1A). He had no medical history of bronchial asthma, atopic dermatitis, allergic rhinitis, history of steroid treatment, infection with human immunodeficiency virus (HIV), hepatitis B virus (HBV), or hepatitis C virus (HCV), sarcoidosis, or administration of immunosuppressant drugs. However, he had a medical history of generalized itchiness without any known cause.

Physical findings showed no fever (body temperature, 36.3°C) and no dry mouth; the patient's unstimulated (7.8 mL/15 min) and stimulated (5.66 g/2 min) salivary flow rates were normal. Diagnostic imaging modalities, including ultrasonograms (US), computed tomography, and magnetic resonance imaging (MRI), are commonly used for KD. On US, left PG showed reticular pattern of hypoechoic areas with high vascularization (Fig. 1B) and enlarged lymph nodes. MRI showed remarkable swelling of left PG with marked enhancement (Fig. 1C). Similar lesion was also seen in the subcutaneous area of the dorsal portion of right PG (Fig. 1C).

Laboratory findings showed an increased eosinophil percentage (29.5%). Other serum chemistry parameters were within normal limits. Immunological tests were negative for antinuclear antibody, anti-SS-A antibody, anti-SS-B antibody, and rheumatoid factor. Serum levels of IgG, IgA, IgM, and IgG4 were within normal limits (1039 mg/dL, 237 mg/dL, 66 mg/dL, and 96.8 mg/dL), but serum IgE concentration was elevated (14,834 U/mL). Serum soluble interleukin-2 receptor concentration was normal (562 U/mL).

**Figure 2:**
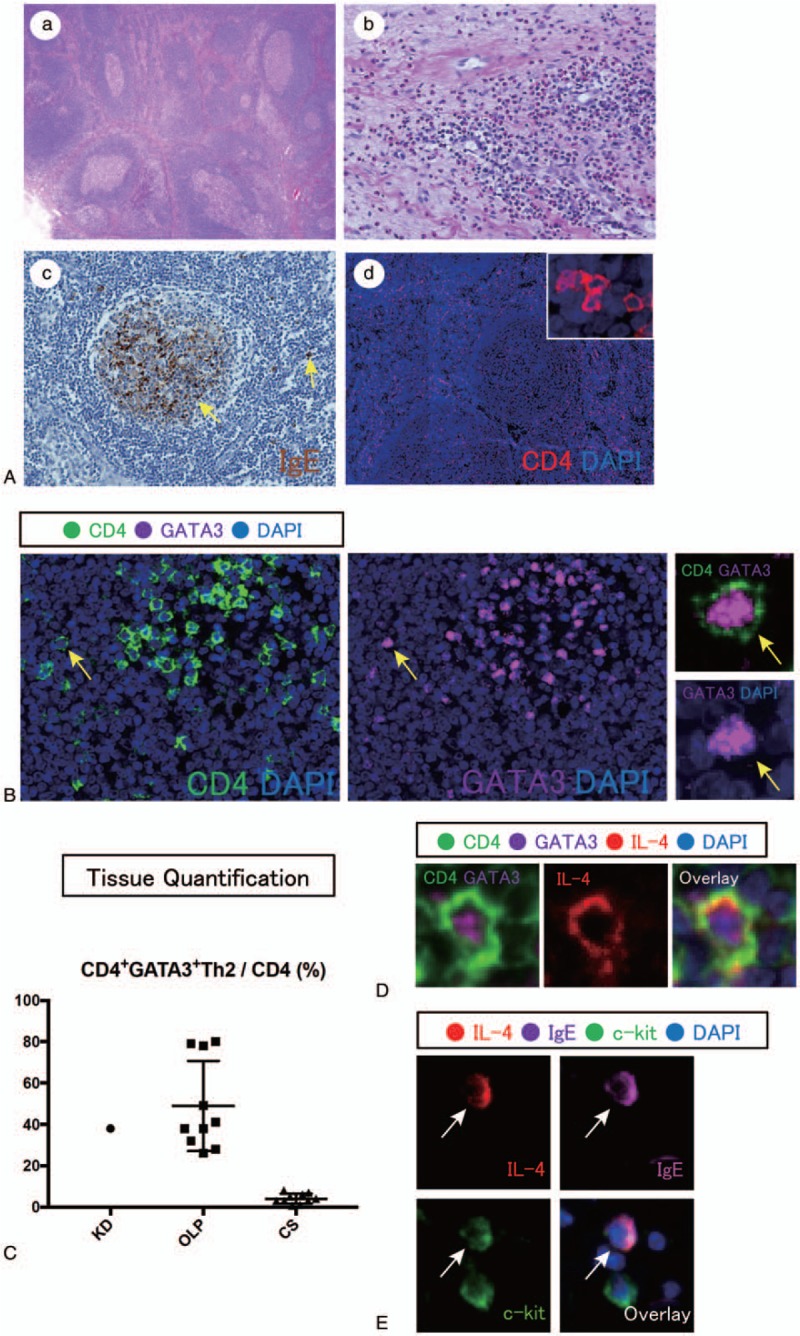
(A) (a) Low-power view of tertiary lymphoid organs with ectopic germinal centers formation in parotid glands from the patient in this report (hematoxylin and eosin, H&E). (b) High-power magnification of the fibrotic area showing numerous plasma cells and eosinophils (H&E). (c) Immunohistochemical staining for IgE in ectopic germinal centers. (c) Immunofluorescence staining of CD4 (red) and DAPI (blue) in parotid gland from the patient in this report. (B) Multi-color immunofluorescence staining of CD4 (green), GATA3 (magenta) and DAPI (blue) in parotid glands from the patient in this report. (C) Quantification of CD4+ GATA3+ T cells and CD4+ T cells in the parotid glands of the patient in this report (KD), compared with those cells in 10 buccal mucosas from patients with oral lichen planus, and 10 salivary glands from patients with chronic sialoadenitis. (D) Multi-color immunofluorescence staining of IL-4 (red), CD4 (green), GATA3 (magenta), and DAPI (blue) in parotid glands from the patient in this report. (E) Multicolor immunofluorescence staining of IL-4 (red), c-kit (green), IgE (magenta), and DAPI (blue) in parotid glands from the patient in this report. DAPI = 4′,6-diamidino-2-phenylindole, IgE = immunoglobulin E, IL = Interleukin.

Biopsy of enlarged lymph nodes showed no evidence of specific inflammation or malignancy. Biopsy of affected PG showed numerous tertiary lymphoid organs (TLOs) containing germinal centers (Fig. 2A-a). Expansion of the interfollicular area was observed, containing large numbers of eosinophils, along with plasma cells and lymphocytes. Affected PG demonstrated eosinophilia (Fig. 2A-b).

For immunohistochemical analysis, 4-μm formalin-fixed and paraffin-embedded sections were prepared and stained. IgE-positive cells were present in germinal centers of secondary lymphoid organs (SLOs) and TLOs (Fig. 2A-c). Some IgE-positive cells were present outside of those germinal centers. Thus, based on the clinical trial of subcutaneous painless mass in the head and neck region, combined with eosinophilia and elevated serum IgE level, the patient was diagnosed with KD.

For multicolor immunofluorescence staining, tissue samples were fixed in formalin, embedded in paraffin, and sectioned. These specimens were incubated with antibodies: CD4 (clone: CM153A; Biocare, Pacheco, CA), GATA3 (clone: CM405A; Biocare), IL-4 (clone: MAB304; R&D System, Minneapolis, MA), c-kit (clone: LS-A9389; LSBio, Seattle, WA) and IgE (clone: MHE-18; BioLegend, San Diego, CA) followed incubation with secondary antibody using an Opal^TM^ Multiplex Kit (Perkin Elmer, Hakataeki Higashiku, Hkata-ku Fukuoka, Japan). The samples were mounted with ProLong^TM^ Diamond Antifade mountant containing 4′,6-diamidino-2-phenylindole (DAPI) (Invitrogen, minato-ku, Tokyoto, Japan). Images of the tissue specimens were acquired using the TissueFAXS platform (TissueGnostics, Los Angeles, CA). For quantitative analysis, the entire area of the tissue was acquired as digital greyscale images in 5 channels with filter settings for Fluoresceinisothiocyanate isomer-I, Cy3 and Cy5 in addition to DAPI. Cells of a given phenotype were identified and quantitated using the TissueQuest software (TissueGnostics), with cut-off values determined relative to the positive controls. This microscopy-based multicolor tissue cytometry software permits multicolor analysis of single cells within tissue sections similar to flow cytometry.

**Figure 3:**
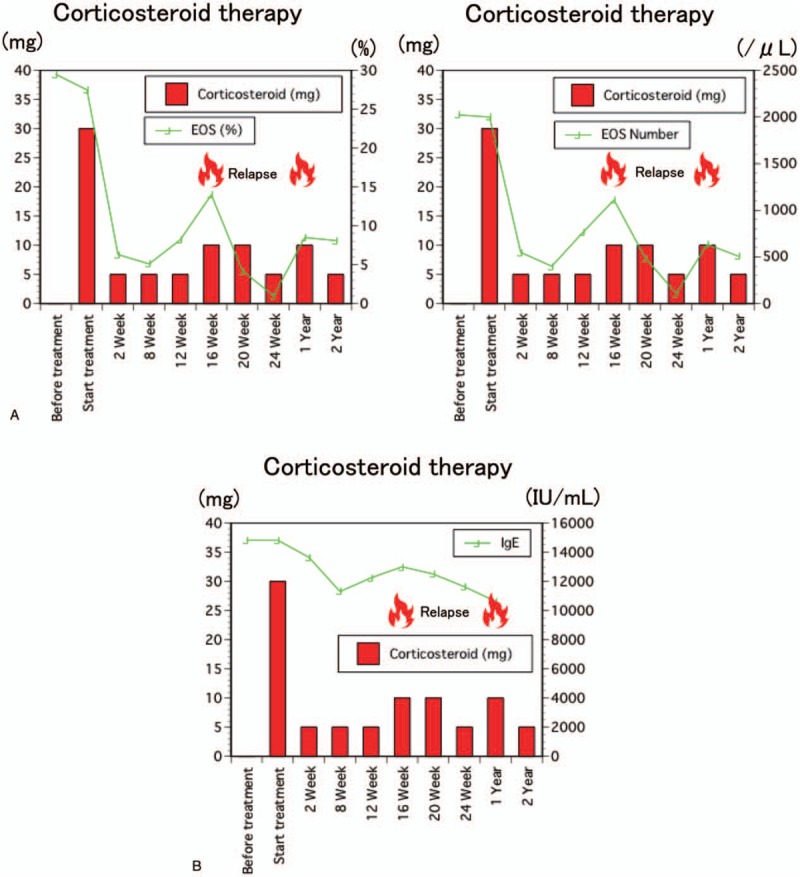
(A) Serial changes in the percentages and counts of eosinophil after corticosteroid therapy. (B) Serial changes in the IgE levels after corticosteroid therapy. IgE = immunoglobulin E.

Immunofluorescence revealed infiltrating CD4+ T cells in affected PG (Fig. 2A-d); CD4+ T cells are abundant, in particular, CD4+ GATA3+ T cells were abundant in affected tissues (Fig. 2B). Multicolor staining analyses revealed that approximately 40% of CD4+ T cells in this patient expressed GATA3 (Fig. 3).

As comparative subjects, 10 patients with Oral lichen planus (OLP) (5 men and 5 women; mean ± standard deviation age, 55.3 ± 9.0 years) who were referred to the Department of Oral and Maxillofacial Surgery, Kyushu University Hospital between 2005 and 2018 were included in the study. Medical records were retrospectively reviewed after the diagnosis. There was no documented history of HIV, infection with HBV, HCV, sarcoidosis, or any other known immune-depressants for any patient. CD4+ GATA3+ T cells were abundant in patients with KD and OLP rather than those of chronic sialoadenitis (CS). Some CD4+ GATA3+ T cells expressed interleukin (IL)-4 in affected tissues with KD (Fig. 2D). IL-4+ IgE+ ckit+ mast cells were also detected in affected tissues with KD (Fig. 2E).

The patient underwent corticosteroid therapy (30 mg/d) and had been followed for 2 years. The mass decreased in size, beginning in the second week of corticosteroid therapy; moreover, skin itchiness decreased after therapy was initiated. Eosinophil counts in the blood gradually decreased after therapy (Fig. 3A). However, high serum levels of IgE persisted after therapy (Fig. 3B).

## 3 Discussion

KD was first described as eosinophilic hyperplastic lymphogranuloma by Kim and Szeto in 1937 in China. In 1948, Kimura et al described the pathological features of this condition, and since then KD has become widely recognized; notably, it is predominantly observed in East Asian male patients.^[1]^ The characteristic histopathologic findings of this patient were matched, infiltration of eosinophils, follicular hyperplasia with well-formed mantle zones, IgE reticular staining in GC, as previous report.^[1]^

Elevated serum IgE, peripheral blood eosinophilia, and IgE-positive B cells in germinal centers are present in KD, suggesting that these are associated with an allergic response. Antigen-specific B cells are toward to IgE secreting B cells, skewed to IgE class switching. Notably, germinal centers are important sites of B-cell selection, where B cells undergo numerous somatic hypermutations and class-switching. IgE is found in B cells both inside and outside of germinal centers in SLOs and TLOs in KD.

CD4+ Th cells subset can be characterized by its ability to sense different inductive cytokines, program the expression of distinct transcription factors, and function by producing select cytokines and chemokine receptors to best control specific pathogens or prevent immune pathology.^[5]^ CD4+ GATA3+ Th2 cells might play important roles in IgE production through IL-4 and IL-5, as well as activation of eosinophils. The presence of Th2-type cytokines and chemokines (eg, IL-4, IL-5, Regulated on activation normal T cell expressed and secreted [RANTES], and Chemokine (C-C motif) lingand 18 [CCL18]) within KD lesions suggests that this disease may be caused by Th2 cells.^[6]^ However, there has been no direct evidence to implicate Th2 cells in affected lesions from patients with KD. Notably, we reported that Th2-mediated inflammation might contribute to the pathogenesis of OLP.^[7]^ We also reported that CD4+ GATA3+ Th2 cells are sparse in affected lesions with CS.^[4]^ Here, we showed direct evidence for the existence of CD4+ GATA3+ T cells in affected tissues from a patient with KD. Additional research for CD4+ follicular helper T (Tfh) cells is needed to further elucidate the pathogenesis of KD. Importantly, Tfh cells also aid B cells during T-dependent immune responses; Tfh cells are essential for germinal center formation, affinity maturation, and the development of most high-affinity antibodies and memory B cells.^[8]^ Thus, Tfh cells could be involved in the pathogenesis of KD.

Presumably, IgE+ mast cells, activated via high-affinity IgE receptor, may also be found outside germinal centers. Kimura et al^[6]^ reported that mast cells may play a role in the synthesis of IgE and eosinophilic infiltration associated with KD through the expression of key cytokines/chemokines (eg, IL-4, IL-5, eotaxin, and RANTES). Mast cells play an important role in IgE-mediated allergic diseases and have been shown to produce multifunctional cytokines. In this case, we found that IgE+ c-kit+ activated mast cells expressed IL-4 in affected lesions in a patient with KD. However, IL-4+ CD4+ GATA3+ T cells and IL-4+ Tfh cells were sparse in affected lesions in this patient (data not shown), suggesting that residential IgE+ activated mast cells in affected lesions might be major sources of these cytokines. However, additional studies of mast cells in KD are needed to confirm this hypothesis.

Although various therapeutic modalities have been used, there is no established treatment for KD. The most common therapies include surgical excision or radiotherapy of enlarged masses, as well as administration of local or systemic corticosteroids and immunosuppressive agents. However, the masses tend to recur after treatment. Surgery followed by low-dose radiation therapy appears to result in the longest period of remission.^[9]^ Steroid therapy can shrink the mass, but it often re-enlarges after treatment. In our case, the patient experienced relapse, even with the application of corticosteroid therapy. High serum IgE levels persisted after corticosteroid therapy, suggesting that IgE-secreting B cells might be long-lived in patients with KD. Additionally, peripheral blood eosinophilia served as a marker of recurrent disease in this case.

In conclusion, we have described a Japanese man who exhibited KD. The cause of KD is unknown but may result from an abnormal immune response. Although this is a case report, interactions among expanded IgE+ B cells, CD4+ GATA3+ T cells, eosinophils, and activated mast cells play a critical role in the pathogenesis of KD, suggesting that effective treatment for KD would be to suppress the abnormal immune response. However, additional research regarding disease-specific immune cells is needed to further elucidate the pathogenesis of KD.

## Acknowledgment

The authors thank Ryan Chastain-Gross, PhD, from Edanz Group (www.edanzediting.com/ac) for editing a draft of this manuscript.

## Author contributions

**Conceptualization:** Takashi Maehara.

**Data curation:** Takashi Maehara, Ryusuke Munemura, Noriko Kakizoe, Naoki Kaneko.

**Formal analysis:** Takashi Maehara, Ryusuke Munemura.

**Funding acquisition:** Takashi Maehara.

**Investigation:** Takashi Maehara, Ryusuke Munemura, Naoki Kaneko, Yuka Murakami, Naoki Kaneko

**Methodology:** Takashi Maehara, Ryusuke Munemura, Naoki Kaneko

**Project administration:** Takashi Maehara.

**Resources:** Takashi Maehara, Noriko Kakizoe.

**Software:** Takashi Maehara, Yuka Murakami, Moriyama Masafumi.

**Supervision:** Takashi Maehara, Seiji Nakamura.

**Validation:** Takashi Maehara, Yuka Murakami, Tamotsu Kiyoshima, Shintaro Kawano

**Visualization:** Takashi Maehara, Ryusuke Munemura

**Writing – original draft:** Takashi Maehara.

**Writing – review and editing:** Takashi Maehara, Mayumi Shimizu, Tamotsu Kiyoshima, Seiji Nakamura.
